# Postoperative Aseptic Intracranial Granuloma: The Possible Influence of Fluid Hemostatics

**DOI:** 10.1155/2012/614321

**Published:** 2012-08-09

**Authors:** Mario Ganau, Nicola Nicassio, Leonello Tacconi

**Affiliations:** Department of Neurosurgery, University Hospital Trieste, Strada di Fiume 447, 34149 Trieste, Italy

## Abstract

*Background.* Numerous reports have demonstrated how postoperative intracranial granulomas can often mimic neoplasm clinically, radiologically, and even macroscopically. Herein we present an unusual case of postsurgical intracranial aseptic granuloma secondary to a chronic inflammatory reaction without any identifiable retained foreign body. *Case Description*. A 71-year-old patient started complaining of severe headache seven months after surgical excision of WHO Grade I right frontal falx meningioma. CT and MRI scans disclosed a contrast-enhanced lesion with diffuse mass effect in the previous surgical site. The lesion was resected; intraoperative finding and histological specimens led to the diagnosis of postoperative granuloma, likely expression of a glial reaction to the fluid absorbable hemostatics applied in the surgical site after meningioma excision. The possible granuloma-inducing materials and the timing of granuloma formation are discussed. *Conclusion*. A comprehensive analysis of clinical and neuroradiological data, as well as results of blood tests including positive and negative acute phase proteins, is mandatory to raise the suspicion of postoperative granuloma. The treatment options should be evaluated on a case-by-case basis, with a conservative attitude being the one of choice only for patients without progressive neurological deficit. Alternatively, aggressive surgical treatment and histopathological examination should be advocated.

## 1. Introduction

Intracranial granulomas are a rare pathologic finding; their formation is expression of chronic inflammation characterized by accumulation of modified macrophages and is initiated by a variety of infectious and noninfectious agents [1]. Infectious granulomas are the most frequent, generally related to tuberculosis or sarcoidosis. Noninfectious granulomas are instead a reaction to a foreign body. The latter are reported to happen anywhere from months to decades after surgical procedures, and numerous reports have demonstrated how granulomas can often mimic neoplasm clinically, radiologically, and even grossly [2].

In this paper we present an unusual case of delayed intracranial granuloma, occurring seven months after total resection of a right frontal falx transitional meningioma (WHO Grade I). The mass mimicking a relapse of tumor growth was microscopically excised: interestingly, histological examination showed a chronic aseptic inflammatory process inconsistent with tumor recurrence, abscess, or plasma cell granuloma. After a revision of all the clinical and radiological data and supported by the fact that no evidence of foreign body was histologically found into the granuloma, but elements of advanced degradation of the fluid absorbable hemostatics applied in the surgical site after the meningioma excision, we have speculated that this space-occupying mass might likely result as a glial reaction to their intraoperative use.

Identification of postoperative granulomas is important to prevent inappropriate treatment of presumed tumor recurrence. A discussion of the relevant literature is provided with special attention to possible granuloma-inducing materials, diagnostic flow chart, and therapeutic options.

## 2. Case Report

**Figure 1:**
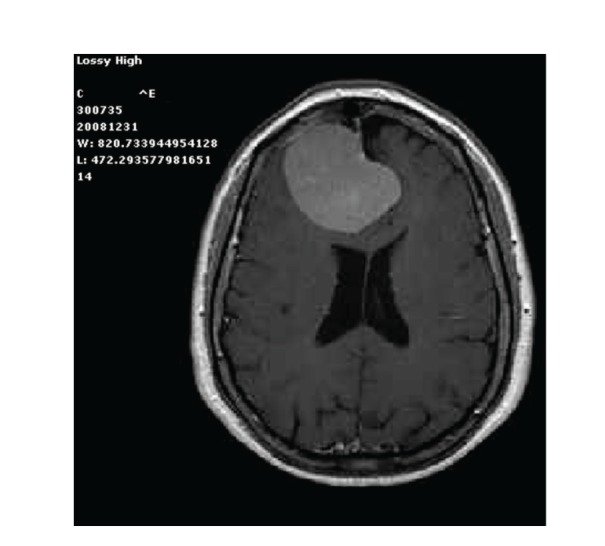
Preoperative contrast-enhanced axial MRI of the right frontal falx meningioma.

A 71-year-old woman was rehospitalized in our Neurosurgical Department with an irregular intracranial contrast enhanced lesion, seven months after a craniotomy for removal of a frontal falx meningioma (Figure 1).

Anamnesis was positive for hypertension, diabetes, poliallergic asthma, Hashimoto's thyroiditis, and bladder carcinoma. At the time of the first admission the frontal lesion was responsible for specific symptoms such as mood disturbances, insomnia, and headache. Surgical excision was then proposed and, after desensibilization therapy (antihistaminics and corticosteroids administered for three days prior to the scheduled intervention), performed through a right frontal craniotomy with removal of the infiltrated falx by standard technique with ultrasonic surgical aspirator. Hemostasis was obtained by filling part of the surgical cavity with absorbable hemostatics such as bovine-derived gelatine and human-derived thrombin matrix (Floseal, Baxter) and oxidized regenerated cellulose (Surgicel, Ethicon Inc.); finally, a dural graft implant (Duraform, Codman) was used to replace the calvarian dura mater removed, in order to achieve a Simpson Grade I excision.

Her postoperative course was uneventful; as well the early CT and MRI scans were unremarkable, confirming the complete excision of the tumor. Blood tests revealed an unspecific increase of the high-sensitivity C-reactive protein (hs-CRP) levels above the range of normality (72,9 mg/L; normal value <5 mg/L), but this data was not associated with fever, leucocytosis, alteration of seric procalcitonin, or other signs of flogosis. Given the histological diagnosis of WHO Grade I transitional (mixed) meningioma (Figure 4) and good clinical conditions the patient was discharged home and enrolled in our standard follow-up program.

**Figure 2:**
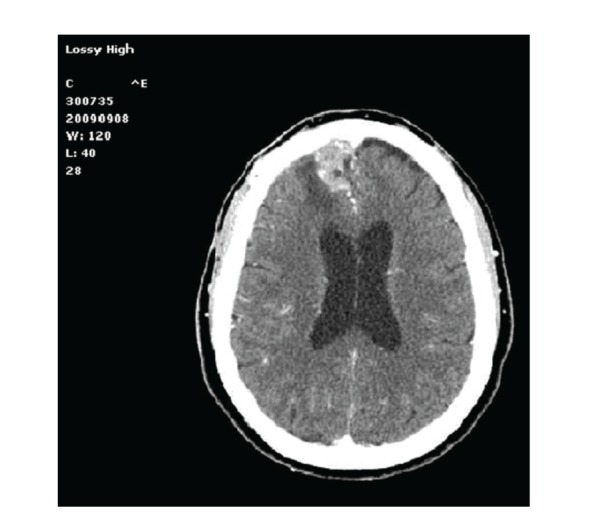
Contrast-enhanced axial CT scan of the postoperative intracranial granuloma.

**Figure 3:**
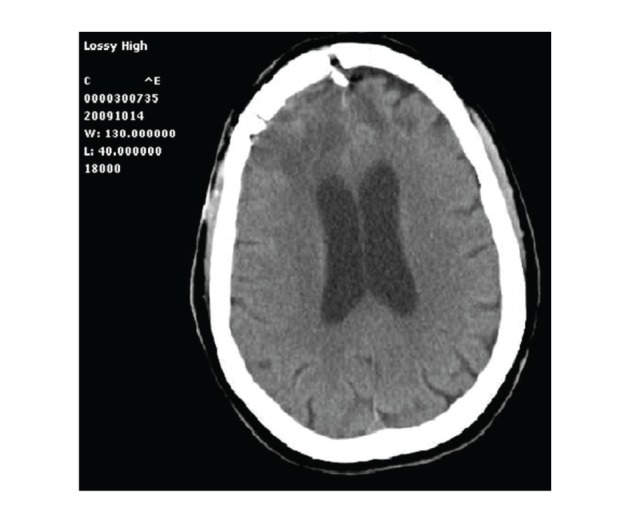
Axial CT scan few days after granuloma excision: note the dramatic but incomplete reduction of brain edema.

**Figure 4:**
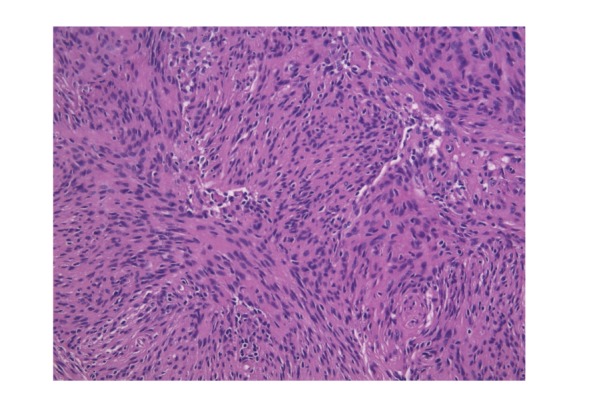
Hematoxylin-Eosin staining. A sample from the first operation is shown. It is possible to appreciate spindle-shaped cells resembling fibroblasts on a matrix abundant in collagen and reticulin. The final diagnosis was WHO Grade I transitional (mixed) meningioma.

**Figure 5:**
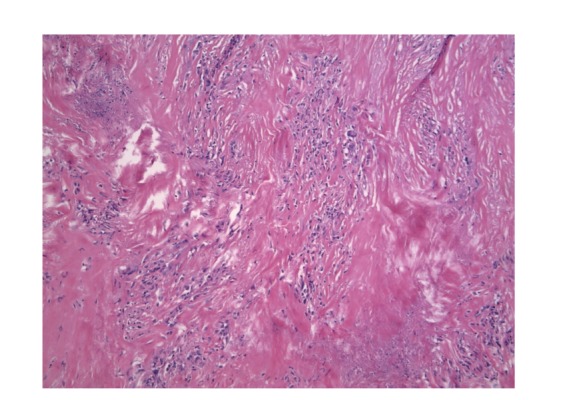
Hematoxylin-Eosin staining. A histological sample from the second operation is shown. Cells are less represented and not well ordered as in Figure 4. There are many acellular zones.

**Figure 6:**
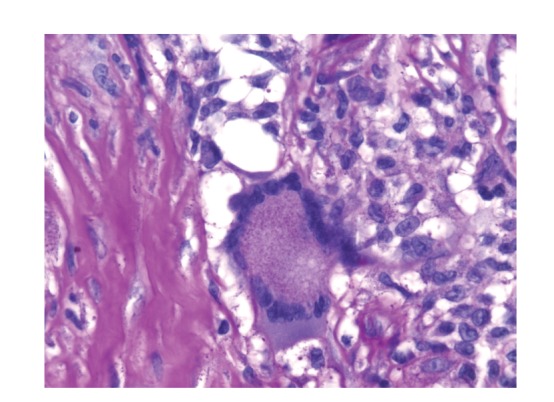
Hematoxylin-Eosin staining. A giant multinucleate cell, typical element of postoperative granuloma, is shown.

Seven months later she was referred back by her General Practitioner for persistent headache. The surgical wound was completely healed; there was no evidence, nor history of fever, but hs-CRP levels kept being slightly higher than normal range values (59,4 mg/L). A cephalorachidian liquor (CSF) analysis was then performed by a lumbar puncture, but resulted completely normal both chemically and microbiologically. The CT scan of the brain revealed along the right frontal falx an irregular 2.5 cm contrast-enhanced lesion, associated with surrounding edema responsible for diffuse mass effect (Figure 2); on MRI scan this mass was isointense in T1 and hyperintense in T2, DW and T-FLAIR sequences. Relapse of tumor growth, even if unlikely, was suspected; therefore a reopening of the previous frontal craniotomy was promptly scheduled. Upon dural opening the mass appeared grayish, irregular, and loosely adherent to the surrounding brain tissue, resulting immediately inconsistent with the preoperative diagnosis of meningioma recurrence. Intraoperatively, both a cytological analysis and further confirmation on frozen section examination led to a diagnosis of gigantocellular flogosis. The removal was microscopically total, and all the specimens obtained were sent to the Pathology Department for histological analysis: no evidence of atypical mitosis was found, while sclerotic tissue due to a diffuse and dense inflammatory infiltrate, rich in foamy macrophages and mononuclear cells, was detected on H & E stain (Figures 5 and 6). The absence of polyclonal plasma cells and lymphocytes ruled out the suspicion of plasma cell granuloma; hence a conclusive histopathological diagnosis of postoperative granuloma was made even if no evidence of foreign body was detected, and special stains, such as Giemsa, Grocott, PAS, and AFB, for bacteria (including acid fast organisms) and fungi, resulted negative. A microbial culture in agarose gel medium, performed at the time of operation, resulted negative too. The first postoperative week was characterized by a successful regression of headache; even though an early CT scan revealed the persistence of a brain oedema surrounding the surgical site, further neuroradiological followup disclosed its gross but incomplete resolution (Figure 3). The patient was followed up on an outpatient basis; one year after the second surgery, she is neurologically negative without any other clinical complaint.

## 3. Discussion

Postoperative intracranial granulomas are luckily rare. A Medline search of the literature published until December 2011, using subject heading “intracranial postoperative granuloma,” produced only 42 results. Most of these articles describe aseptic foreign body granuloma formation caused by a variety of substances including gel foam [3], surgical swab [4], bone wax [5], cotton pledgets [6, 7], rayon [8], suture [9], oxidized cellulose [10], microfibrillar collagen [11], muslin gauze [12], and polytetrafluoroethylene [13]; some others refer also to infectious granulomas secondary to aneurism surgery [14] or gasserian ganglion decompression [15]. Furthermore, intracranial granulomas not related to previous neurosurgical procedures may have a tumor-like origin, as in case of plasma cell granulomas [16] and neurosarcoidosis [17], or infectious aetiology, associated or not with extracranial localizations, generally due to fungal [18] or acid-fast organisms [19]. Thereafter, the case presented in this paper is particularly interesting since the granuloma formation was secondary to a postsurgical aseptic chronic inflammatory reaction without any identifiable retained foreign body but elements of advanced degradation of the fluid absorbable hemostatics applied in the surgical site after the meningioma excision.

Despite the location and the surrounding oedema, suggestive for parenchymal invasion, a recurrence of the meningioma was thought to be unlikely because of three reasons: (1) the Simpson grade I excision obtained, (2) the histological low grade at first surgical removal, and (3) the short delay between the two hospitalizations. Furthermore, the hypothesis of infection was not supported by the anamnesis, since surgical wound was originally classified as Class I according to CDC Surgical Wound Classification, nor by any clinical signs or symptoms. Moreover, the negativity of procalcitonin firstly, and CSF examination secondary, seemed to controvert the elevation of hs-CRP. Although we had many concerns regarding the possible diagnosis, the degree of the surrounding edema contributed to prompt exploration via reopening of the previous frontal craniotomy: the lesion was inconsistent with a meningioma recurrence or with tumor-like growth, and no foreign body or exudates were found to support the suspicion of abscess. The histopathological examination was the only tool allowing for a definitive diagnosis of postoperative granuloma; at that time, despite the presence of some mononuclear cells within the specimen, the differential diagnosis of plasma cell granuloma was definitely excluded by the absence of polyclonal proliferation of mature plasma cells. After a reanalysis of the surgical technique used for the removal of the falx meningioma, we argued that the granuloma formation was most likely due to the amount of absorbable hemostatics used to fill the surgical cavity at the time of the first surgery.

A variety of local hemostatics including absorbable gelatin sponge, collagen, and oxidized cellulose are commercially available; their application is recommended when cautery, ligature, or other conventional procedures are ineffective or impractical. Proper handling of absorbable hemostatic agents is essential to control bleeding, and even if the hemostat is expected to dissolve promptly, when it is retained in or near bony or neural spaces, the minimum amount should be left in place after hemostasis is achieved. Generally, topic hemostatics are reabsorbed within a couple of weeks, and during this period minimum inflammation without strong foreign body reactions or blockade of healing is possible; rarely, strong foreign body reactions, chronic inflammation, and infections can cause granuloma formation after their use [20]. Although the incidence of postoperative granulomas secondary to the use of hemostatics is well recognized, only few authors have reported their occurrence after neurosurgical procedures [3, 10, 11, 21, 22]. Risks of foreign body reaction or mass effect may be different from agent to agent: in fact whereas a dry local hemostat absorbs body fluid of several times its own weight and expands postoperatively, the maximum swell volume of fluid hemostats is reported to be about 20%, and it is achieved within 10 minutes (Floseal hemostatic matrix instructions to use). Microfibrillar collagen, for instance, has been responsible for some cases of huge granulomas in general surgery [23, 24]; on the other hand, bovine-derived gelatine and human-derived thrombin are considered among the safest, because absorption occurs within 6 to 8 weeks, consistent with normal wound healing [20]. Indeed, among the adverse events deemed to be possibly related to the use of such a fluid hemostats are infection (6% of cases) and local inflammation (with a rate lower than 1%) (Floseal, Hemostatic Matrix Instructions). Hemostatics may also induce allergic reactions, but generally those depend on the antigenicity of the agent used: the incidence of positive reactions to collagen is reported as 3% [20] while for gelatin is less than 1%, even in people sensitive to bovine materials (with a rate as low as 0,01%) (Floseal, Hemostatic Matrix Instructions).

It is generally accepted that by using local hemostats, it is possible to improve the condition of the patient, reduce complications, and lower direct and indirect costs, but it is necessary to stress the point that documentation is important with regard to the hemostat used, including the name of the agent, site, and amount: that information may be useful as a reference in the interpretation of postoperative diagnostic images changes [20]. Postoperative granulomas are reported to occur after several months or even years from surgery; postoperative radiographic changes may thus create diagnostic doubts, especially in neurooncology, because they can mimic tumor recurrence [11, 22, 25–27]. To this regard Feldman et al. [2] suggested that MRI findings of low intensity T1-weighted with heterogeneous high intensity on T2-weighted images, respectively, seem to be the rule for intracranial postoperative granulomas. In such cases, it is well known that retained hemostatics may sometimes mimic an abscess, and the case described here confirms that on similar cases DW images could be useful in distinguishing between granulomas and exudates, as previously reported [2, 8]. It has also been mentioned that the hemostatic-related glial inflammation may be the primary responsible for hyperintensity on T2-weighted and FLAIR MRI images [6, 10], and our case is exemplificative of the dramatic vascular/cytotoxic oedema that can result from a postoperative granuloma of such genesis.

Given the possibility of a multifactorial process (i.e., the autoimmune trait or the poliallergic condition of our patient could have played a role in the granuloma formation), that may also be self-limited in certain individuals, the success rate of treatment is equally difficult to determine. Surgical exploration and lysis of adhesions, corticosteroids, antibiotics, and conservative observation have all been attempted. Some reports confirm that symptoms may improve after corticosteroid administration, but others suggest that this correlation might be questionable; nevertheless the failure of a high dosage 3-to-6-week course of steroids should lead to surgical reexploration.

The review of the literature confirms that despite the widespread use of fluid hemostatics in thousands of operations performed by neurosurgeons each year, this is not a well-recognized outcome. Any proposal for a diagnostic flow chart is merely difficult, since history or signs of flogosis may be lacking; consequently a comprehensive analysis of clinical (wound healing, body temperature, etc.) and neuroradiological data, as well as results of blood tests including positive (CRP, fibrinogen, ferritin) and, when available, negative (albumin, transferrin) acute phase proteins is mandatory to raise the suspicion. Thus, it seems reasonable that treatment options should be evaluated on a case-by-case basis, with a conservative attitude being the one of choice only for patients without progressive neurological deficit; in the other cases an aggressive surgical treatment and histopathological examination should be considered in order to guarantee an accurate diagnosis and definitive cure.
